# Organic Sonosensitizers-based SDT with enhanced ROS generation

**DOI:** 10.1016/j.ultsonch.2025.107625

**Published:** 2025-10-15

**Authors:** Qianyun Shan, Rumei Li, Bin Ying, Wei Zhu, Xiaojin Wu, Shouxing Xu, Xuanxuan Zhang, Zhikang Xu, Xinyue Zhu, Weiyu Chen, Kai Zhang, Jian Chen

**Affiliations:** a Department of Ultrasound in Medicine, The Fourth Affiliated Hospital of School of Medicine, and International School of Medicine, International Institutes of Medicine, Zhejiang University, Yiwu 322000, China; b School of Chemistry and Chemical Engineering, Zhejiang Sci-Tech University, Hangzhou 310018, China; c Department of Respiratory and Critical Care Medicine, Center for Oncology Medicine, The Fourth Affiliated Hospital of School of Medicine, and International School of Medicine, International Institutes of Medicine, Zhejiang University, Yiwu 322000, China; d Zhejiang Key Laboratory of Precision Diagnosis and Treatment for Lung Cancer, Yiwu 322000, China

**Keywords:** Sonodynamic therapy (SDT), Organic sonosensitizers, O_2_-delivery strategies, Reactive oxygen species (ROS)

## Abstract

Organic sonosensitizer-based sonodynamic therapy (SDT) is an emerging, non-invasive strategy for cancer treatment, leveraging ultrasound (US) activation to trigger reactive oxygen species (ROS) production and induce tumor cell apoptosis. However, the clinical translation of SDT is limited by two key factors: the intrinsically low ROS quantum yield of many organic sonosensitizers and the hypoxic tumor microenvironment (TME), which restricts O_2_-dependent ROS generation. This review systematically examines recent molecular design strategies aimed at enhancing ROS production, including heavy atom incorporation, donor–acceptor (D-A) architecture design, π-conjugation extension, and solubility modulation. Furthermore, we evaluated innovative O_2_-delivery/generation tumor reoxygenation approaches for enhanced SDT, such as O_2_-nanocarriers, in-situ catalytic O_2_ generation, and mitochondrial respiration modulation etc. Notably, integrating clinically validated sonosensitizers like porphyrins with translational O_2_-delivery systems such as perfluorocarbon (PFC) nanoemulsions or vascular normalization, offers a synergistic strategy to overcome tumor hypoxia, amplify ROS generation, and unlock the full therapeutic potential of SDT in future clinical applications.

## 1 Introduction

**Fig. 1:**
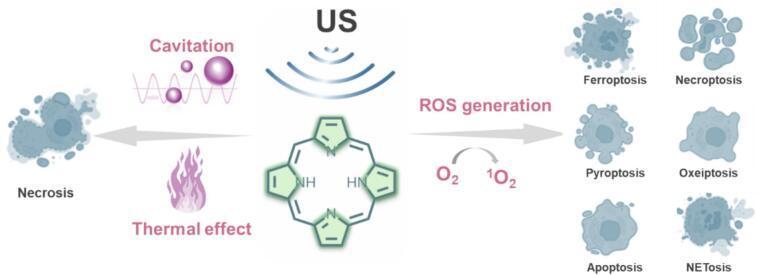
Schematic diagram of SDT mechanisms.

SDT leverages sonosensitizers activated by US to induce oxidative stress within cancer cells, ultimately leading to tumor ablation[1]. This approach offers several unique advantages, including its noninvasive nature, suitability for treating deep-seated tumors, and precise tumor targeting through selective US activation sites[[2], [3], [4], [5]]. The primary mechanisms of cell death in SDT involve mechanical and thermal effects (Fig. 1)[[6], [7], [8]]. Acoustic cavitation, the formation, growth, and collapse of bubbles under US irradiation, would produce shear forces and shock waves and result in cell death[9]. Meanwhile, the absorption and transformation of US mechanical energy could induce hyperthermia in tumor solid and lead to cell necrosis[10]. Most importantly, indirect cytotoxicity mediated by the generation of ROS by sonosensitizers, such as singlet oxygen (^1^O_2_) and hydroxyl radical (•OH) converted from O_2_ in tumor, is the crucial mechanism of SDT[11].

ROS accumulation induced oxidative stress is a key factor in SDT efficacy, contributing to regulated cell death[[12], [13], [14], [15]], manifested by ferroptosis, pyroptosis, apoptosis, oxeiptosis or NETosis (Fig. 1)[16]. Intracellular ROS can initiate lipid peroxidation, which compromises mitochondrial membrane integrity and results in mitochondrial membrane potential depolarization with increased permeability, ultimately triggering apoptosis and necrosis[17,18]. Moreover, mitochondrial damage facilitates the release of cytochrome *C* into the cytoplasm, where it activates the caspase-dependent apoptotic pathway, indirectly leading to the tumor cells elimination[19,20]. Further, the release of cellular contents can activate the immune system, initiating a secondary attack against tumor cells[21]. However, the biological effects of ROS are highly concentration-dependent[16,22]. While moderate ROS levels can stimulate cancer cell proliferation and contribute to drug resistance, only excessive ROS stress can induce cancer cell death and enhance SDT[[23], [24], [25]].

Sonosensitizers are crucial agents in SDT, which absorb US energy, transfer it to O_2_ to generate ROS within the TME [3,6,26]. The ROS generation efficiency depends on the structures of sonosensitizers. Compared with inorganic sonosensitizers (e.g., gold [27], silver [28], or iron oxide [29,30] nanoparticles), organic sonosensitizers including aromatic small molecules[31,32] (e.g., porphyrins) and polymeric compounds[33,34] allow tailored design for tumor targeting [35], toxicity reduction[31], and biocompatibility improvements, making them a safer option for clinical applications. Most importantly, structure modification, such as introducing heavy atoms[36], spin-conversion units[37] or extending π-conjugated systems[38] etc. could promote intersystem crossing (ISC) and optimize energy transfer, thus promoting ROS generation efficiency[39].

The O_2_ concentration in tumor solid is essential to SDT efficacy [8,9,[40], [41], [42], [43], [44]]. In tumor solid, O_2_ concentration at 0.02–2 % (below 2.5 mmHg *p*O_2_) is significantly lower than that in normal cells at 2–9 % (40 mmHg *p*O_2_)[[45], [46], [47], [48]]. The hypoxic TME arises from diffusion-limited hypoxia (caused by rapid tumor growth outpacing O_2_ supply) and perfusion-limited hypoxia (resulting from irregular blood flow in tumor vasculature)[49]. The tumor hypoxia resulted in the failure of not only SDT, but also many other O_2_-dependent therapeutic modalities, including chemotherapy[50], photodynamic therapy (PDT) [51] and radiotherapy[52]. Even worse, SDT will constantly deprive the tumor O_2_ and deteriorate the local hypoxic status, which may connect to poor clinical prognosis. Therefore, it is crucial to increase O_2_ concentration in TME to improve cancer SDT efficacy. Though tumor reoxygenation remains a considerable challenge, strategies focusing on tumor-targeting O_2_-delivery and in-situ generation have been developed with considerable therapeutic effectiveness[7,8,53,54].

**Fig. 2:**
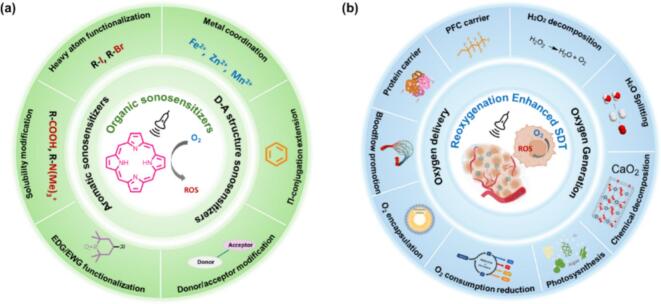
Summary schematic that outlines the sonosensitizer structure modification and tumor reoxygenation strategies to improve SDT efficacy.

In this review, we outline recent advances in both organic sonosensitizer structure modifications and tumor reoxygenation strategies for enhanced SDT, providing elevated ROS generation capability and relieved tumor hypoxia (Fig. 2). The chemical structure modification strategies are classified into six categories, heavy atom functionalization, metal coordination, π-conjugation extension, solubility modification, electron donation group/ withdrawing group (EDG/EWG) functionalization and donor–acceptor (D-A) regulation. Based on mechanism differences, we classified the tumor reoxygenation strategies into O_2_-delivering and in-situ O_2_-generation approaches. We reviewed and analyzed their effectiveness in enhancing SDT efficacy. Building on this foundation, we aim to provide a comprehensive understanding of ROS enhanced SDT and offer practical perspectives for designing novel nanoparticles.

## 2 Organic sonosensitizers

### 2.1 ROS generation mechanisms in SDT

**Fig. 3:**
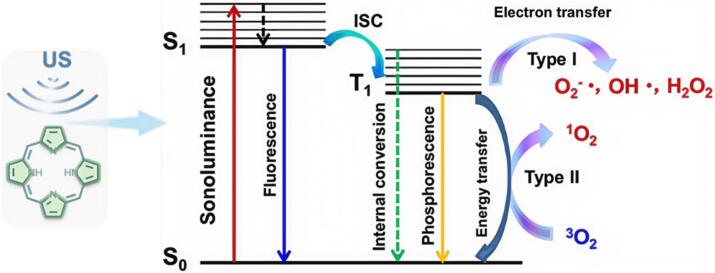
Schematic diagram of ROS generation mechanisms of organic sonosensitizer-based SDT.

When US induces cavitation, the rapid compression and expansion of bubbles generate high pressure (up to thousands of atmospheres) and temperature (potentially exceeding 5,000 K)[55]. High energy release during the collapse of cavitation bubbles transforms acoustic energy into sonoluminescence to transit sensitizers to an excited state[56]. ^1^O_2_ was then generated via type II process that involves energy transfer from excited sensitizer to O_2_ (Fig. 3)[57]. In some circumstances (i.e., D-A sonosensitizers), US energy excites electrons from the highest occupied molecular orbit (HOMO) to the lowest unoccupied molecular orbit (LUMO) producing vigorous intramolecular charge transfer (ICT) to form electron-hole pairs, which would induce type I ROS production (Fig. 3), leading to the formation of various radicals including O₂•⁻, •OH and H₂O₂[58].

ROS generation efficiency of organic sonosensitizers is intricately linked to their structure[31,33,59]. Upon excitation, organic sonosensitizers typically reach the singlet excited state (*S_1_*), which has a short lifetime and often returns rapidly to the ground state with fluorescence emission and non-radiative energy dissipation (Fig. 3). By rational design, the sensitizer can be optimized to promote ISC, a spin-forbidden process that transforms the molecule from *S_1_* to the triplet excited state (*T_1_*)[60]. While the *T_1_*-state is critical for ^1^O_2_ generation due to its longer lifetime and ability to transfer energy to O_2_ efficiently to generate ^1^O_2_[61]. Generally, high ISC rates induce high ROS yield, while the ISC rate is proportional to the energy gap (*ΔE_ST_*) between *S_1_* and *T_1_* states, and *H_SO_* refers to the spin–orbit coupling (SOC) Hamiltonian[62,63]. Therefore, small *ΔE_ST_* and large SOC would promote ISC efficiency, thus enhancing *T_1_*-state sensitizer formation and ROS yield[64]. Additionally, it has been shown that the rate constant for quenching *T_1_*-state sensitizer by O_2_ depends on factors including oxidation potential of the sensitizer, energy of the *T_1_*-state, and polarity of the solvent etc.[[65], [66], [67]]. Therefore, organic sonosensitizers are particularly advantageous in this regard, since their structural features can be fine-tuned to optimize absorption properties, excited state lifetimes, and energy transfer processes, making them versatile tools of SDT.

### 2.2 Types of organic sonosensitizers

**Fig. 4:**
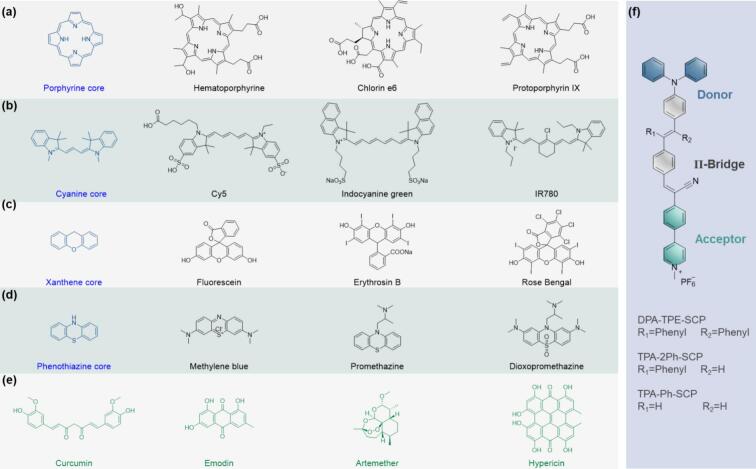
Types of sonosensitizers for cancer SDT.

***Porphyrin derivatives*** have similar 18-π aromatic macrocyclic carbon nucleus structure and exhibit excellent type II ROS generation ability under US irradiation (Fig. 4A)[39]. It is observed that 400–450 nm light produced by 600 kPa, 1.93 MHz US in normal saline solution would activate the porphyrin sonosensitizers and its analog[39]. Commonly used porphyrin-derived sonosensitizers include protoporphyrin IX[68], hematoporphyrin (HP)[69], hematoporphyrin monomethyl ether (HMME)[70], Sinoporphyrin sodium (DVDMS)[71], Chlorine e6 (Ce6)[72], and phthalocyanine[73]. Notably, porphyrin derivative Photofrin has been approved by U.S. Food and Drug Administration (FDA) for its clinical use in lung cancer treatments[74]. The clinical SDT of porphyrin derivatives are still limited by several intrinsic drawbacks, including structural rigidity, poor water solubility, and inadequate tumor targeting. In addition, porphyrins inherently exhibit relatively large *ΔE_ST_*, which hinders ISC efficiency and limits *T_1_*-state formation and ROS generation[75].

***Cyanine derivatives*** have gained attention as potential sonosensitizers due to their strong absorption in the near-infrared region, excellent electron transfer properties, and tunable photophysical characteristics (Fig. 4B)[1]. Cyanine derivatives, particularly Indocyanine Green (ICG, FDA approved), IR-780, Cy5, and Cy7, offer promising features as sonosensitizers. ICG can generate ROS through both Type I and Type II pathways under US, and was widely used in imaging and SDT[76]. IR family (IR-780 & IR-820) showed high lipophilicity and preferential accumulation in tumor mitochondria, making them good sonosensitizers in SDT[77]. Cy5, Cy5.5, and their derivatives showed strong fluorescence emission and efficient ROS production under US, making them useful for fluorescence-guided SDT. However, challenges such as aggregation resulted self-quenching, prone to photo- and US-induced degradation, and poor solubility require chemical modifications and nano-formulation strategies to maximize their efficiency in SDT.

***Xanthene derivatives*** (Fig. 4C), such as Rose Bengal and fluorescein, are another important group of aromatic sonosensitizers[78]. Rose Bengal, a halogenated derivative, is particularly notable for its high ISC rate and ^1^O_2_ production efficiency under US[79]. ***Phenothiazine derivatives*** (Fig. 4D), such as methylene blue and toluidine blue, are well-established sonosensitizers known for their high ROS generation and low cytotoxicity[78]. Methylene blue[80], for instance, is effective in SDT due to its ability to undergo efficient ISC for ^1^O_2_ and •OH generation. Besides, ***fluoroquinolone antibiotics*** including ciprofloxacin and norfloxacin represent a unique class of aromatic sonosensitizers[81]. Their aromatic quinolone core structure enables ROS generation when activated by US. These compounds are particularly interesting because they combine sonodynamic properties with antibacterial activity, making them promising candidates for combating bacterial infections and biofilm-associated diseases. ***Natural products*** with aromatic structures (Fig. 4E), such as curcumin[29], hypericin[82] and emodin[83] etc., also exhibit excellent sonodynamic properties. Curcumin, derived from turmeric, is a polyphenolic compound with extensive conjugation, allowing it to generate ^1^O_2_ under US. Similarly, hypericin from *St. John's Wort* demonstrates high ^1^O_2_ yields and strong tumor-targeting properties, making it a promising sonosensitizer.

***D-A sonosensitizers***, composed of an electron-donating group and an electron-accepting group connected by a π-conjugated bridge (Fig. 4F)[[84], [85], [86]], often exhibit broader absorption spectra, thus versatile for activation across a wide range of sonoluminescence. Additionally, D-A architecture introduces strong ICT and leads to charge-separated excited state upon excitation. These states are characterized by a reduced *ΔE_ST_*, which naturally enhances ISC efficiency[87]. Furthermore, D-A based sonosensitizers exhibit distinct twisted conformations and dynamic molecular rotators[84], allowing precise modulation of radiative and non-radiative decay pathways, as well as ISC. More importantly, the vigorous ICT could promote the generation of type I ROS, which can proceed via electron transfer reactions even in oxygen-deficient environments, making it advantageous for treating hypoxic tumors. Typical D-π-A AIE sonosensitizer was constructed by diphenylamino donor (DPA) and (4-styryl-cyano)pyridinium (SCP) acceptor [84]. The *ΔE_ST_* and ISC rate of the D-π-A sonosensitizer could be effectively tuned by using different linkers, facilitating ROS generation upon US activation.

### 2.3 Modification strategies

ISC rate and the stability of *T_1_*-state are key features for the ROS generation of aromatic sonosensitizers. However, the low ISC efficiency of many aromatic sonosensitizers leads to suboptimal ROS generation and SDT efficacy. Besides, the hydrophobic and rigid planar structure of aromatic sonosensitizers promotes strong π-π stacking, leading to aggregation in physiological environments and low solubility. Therefore, structural modifications are often required to enhance ROS generation. Up to present, strategies including introducing heavy atoms, metal ion coordination, π-conjugation extension, EDG/EWG functionalization, solubility modification and D-A regulation etc. have been developed to increase the ROS generation of organic sonosensitizers.

**Fig. 5:**
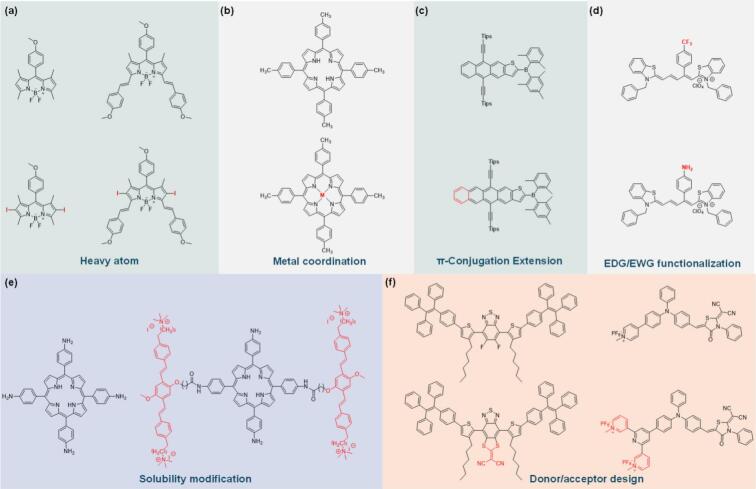
Examples of typical organic sonosensitizer modification strategies, including heavy atom functionalization, metal ion coordination, π-conjugation extension, EDG/EWG functionalization, solubility modification and D-A design[88], [89], [90], [91], [92], [93].

***Introducing heavy atom***. Introducing halogen atoms (i.e., −I, −Br) into the aromatic sonosensitizer structures enhances ISC efficiency and improves ^1^O_2_ generation via the heavy atom effect (Fig. 5a)[94]. Briefly, heavy atoms increase SOC to promote the population of the *T_1_*-state species, thus increasing the energy transfer to O_2_ to generate ^1^O_2_. Heavy atom strategy has been verified in the porphyrin, metalloporphyrin and BODIPY systems[95], [88]. In typical BODIPY system, halogenation not only increased the SOC but also reduced the *∆E_st_*, which would improve the ISC efficiency synergistically (Fig. 5a). Compared with non-halogenation precursors, ^1^O_2_ production of halogenated sensitizer was obviously larger when exposed to both light and US radiation, both in-vitro and vivo[88]. Therefore, excellent SDT efficacy was achieved through intracellular ^1^O_2_ accumulation induced apoptosis in 4 T1 tumor model.

***Metal ion coordination***. Incorporating metal ions into the cavity of aromatic sonosensitizers (i.e. porphyrins) is one of the most effective strategies to increase ^1^O_2_ generation (Fig. 5b). Metal ion coordination could effectively tune the HOMO-LUMO gap of the metalloporphyrin formed to boost the excitation process. Besides, it also enhances ISC by reducing *∆E_st_* and increasing SOC from the metal center, thus facilitating ROS generation[96]. Various metalloporphyrin systems have been proposed, such as zinc[97], palladium[98], and platinum porphyrins[99]. Recently, Cai group systematically compared three metal ion coordinated TTP complexes (MnTTP, ZnTTP, and TiOTTP) encapsulated with human serum albumin[89]. Results found that the lowest HOMO-LUMO gap from MnTTP facilitated the excitation and reduced the *∆E_st_*, making the spin-forbidden ISC process more favorable. Therefore, significantly higher ROS intensity and SDT efficacy were provided by the MnTTP design.

***π-conjugation extension***. Extending the π-conjugation of aromatic sensitizers can improve their sonoluminescence-harvesting capabilities and stabilize their excited states, leading to higher *T_1_*-state yields in type II process. Besides, a large conjugation system could also stabilize the radical intermediates formed in the type I process, thus promoting the radical ROS formation. Liu et al. synthesized boron-doped anthrathiophene (BAnTh) and teranthrathiphene (BTeTh) sonosensitizers (Fig. 5c) and investigated their SDT efficacy[90]. By conjugation extension, BTeTh presents a reduced HOMO-LUMO gap and red-shifted absorptions (λ_Abs_ = 635, 586 nm) at longer wavelengths than BAnTh (λ_Abs_ = 536). Under US irradiation, •OH dominated in ROS generation, which was produced by type I electron transfer process from hydroxyl anions to boron-containing electron-deficient centers. It is reasonable that the large conjugation system could stabilize extra electron and provide better ROS generation efficiency, cell toxicity and SDT efficacy.

***EDG/EWG functionalization.*** By adjusting EDG/EWG groups, the efficiency of Type I and Type II ROS production of sonosensitizers can be optimized for enhanced SDT[100], [101]. Briefly, EDG (e.g., amino or alkoxy groups) tend to reduce *ΔE_ST_* to increase ISC efficiency and improve type II ROS generation. While EWG (e.g., nitro or cyano groups) boost type I ROS production (e.g., O₂•⁻, •OH) by stabilizing charge-separated states and facilitating electron transfer to O_2_[91]. Han et al. developed a pentamethyl cyanine platform utilizing the C2′-aryl substitution with EDG/EWG (Fig. 5d)[91]. The results showed that both ICT and photo induced electron transfer (PET) could be effectively modulated by substitution effect, thereby regulating the ISC efficiency and ROS generation. Specifically, transition from a strong EWG (e.g., –NO_2_) to strong EDG (e.g., –NH_2_), the ICT tendency decreased, while PET tendency increased. This switch improved the overall *T_1_*-state yields, thus promoting the ^1^O_2_ generation and antitumor efficacy. Notably, though the therapeutic effect was tested in a PDT strategy, the mechanism of EDG/EWG-enhanced ROS generation is also applicable to sonosensitizers in SDT.

***Solubility modification****.* Water solubility and aggregation state of sonosensitizers in the vitro/vivo environment affect its SDT efficacy. Aggregation causes self-quenching of the excited states and increases non-radiative decay pathways, preventing ISC and reducing ROS yield. Besides, hydrophobic sonosensitizers have poor bioavailability, limiting their delivery and accumulation at the tumor site. Consequently, maintaining the monomeric state of sonosensitizers through functionalization would result in more efficient ISC and ROS generation[102]. Recently, Jia et al. constructed a new water-soluble porphyrin sonosensitizer by covalently linking water-soluble conjugated oligomers to TAPP (Fig. 5e)[92]. The increased solubility significantly reduced the self-aggregation and boosted intramolecular energy transfer efficiency as well as ROS yield. The excellent ROS generation efficiency under US irradiation led to apoptosis and necrosis of tumor cells. Therefore, increasing the solubility of hydrophobic sonosensitizers is another effective strategy for maximizing their effectiveness in SDT.

***D-A design***. By modifying the donor, acceptor, or conjugated bridge, the D-A design allows for fine-tuning of electronic and photophysical properties. For instance, strong donors (i.e., amines or thiophenes) combined with powerful acceptors (i.e., cyano or nitro groups) can be tailored to achieve desired absorption properties, *T_1_*-state lifetimes, and solubility. Additionally, their asymmetrical structure enhances interaction with biological environments, improving cellular uptake and specificity[87]. In a D-A sonosensitizer[93], by replacing conventional benzothiadiazole with a dithiafulvalene-fused benzothiadiazole acceptor, Zhao et al. provided a new design with twisted conformation. It reduced the HOMO-LUMO gap and optimized the ICT and ISC (Fig. 5f), which not only enhanced ROS generation but also endowed the D-A sonosensitizer with AIE properties. In another A-D-A’ structure sonosensitizer[87], by increasing the cationized pyridine acceptor (Fig. 5f), both *ΔE_ST_* and SOC constant were optimized foreshadowing the optimum efficiency of ISC and ROS generation. Both strategies demonstrate the importance of D-A structure design in improving ROS yield, and SDT efficacy.

Table 1 gives the comprehensive comparison of sonosensitizer modification strategies. Among these, the heavy atom effect and metal coordination strategies are well-suited for aromatic sonosensitizers such as porphyrins, as they enhance SOC and facilitate ISC, albeit with potential concerns related to toxicity and clinical safety. Alternatively, π-conjugation extension, EDG/EWG functionalization and D-A engineering are broadly applicable across various organic sonosensitizer types. These strategies optimize ICT, narrow *ΔE_ST_*, and promote both Type I and Type II ROS production with minimal safety concerns. Furthermore, Solubility modification, particularly through hydrophilic side chains or PEGylation, effectively suppresses aggregation-caused quenching, improving biodistribution and tumor accumulation without compromising ROS generation efficiency. Overall, those based on D-A modulation and solubility control, strike a favorable balance between performance, safety, and clinical feasibility, and thus hold significant promise for next-generation SDT platforms, especially the aggregation-induced emission luminogens (AIEgens) based theranostic sonosensitizers.

**Table 1:** Comparison of sonosensitizer modification strategies.

**Strategy**	**Suitable for**	**Mechanism for Enhancing ROS**	**Advantages**	**Disadvantages**	**Clinical Translation Potential**	**Ref.**
**Heavy atom functionalization**	Aromatic sonosensitizers	SOC enhancement	High ROS efficiency	Heavy atom toxicity	Limited; safety concerns	[88], [94], [95]
**Metal** **coordination**	Porphyrins Phthalocyanines	HOMO-LUMO tune; *ΔE_ST_* reducing; SOC enhancement	Metal imaging contrast	Metal toxicity	Moderate; Metal dependent safety	[89], [96], [97], [98], [99]
**π-conjugation extension**	Most organic sonosensitizers	HOMO–LUMO tune; radical stabilization	Boosts both type I/II ROS	Aggregation & solubility	Moderate; Aggregation concern	[90]
**EDG/EWG functionalization**	Most organic sonosensitizers	HOMO-LUMO tune; *ΔE_ST_* reducing; ICT tune	Versatile	Tedious synthesis	High; Molecular engineering well accepted	[[91], [100], [101]]
**Solubility modification**	Hydrophobic sonosensitizers	Aggregation-induced quenching reducing	Better biodistribution	Activity reduction	High; PEGylation & sulfonation clinically proven	[92], [102]
**D-A** **engineering**	D-A sonosensitizers	HOMO-LUMO tuning; *ΔE_ST_* reducing; SOC enhancement	Additional AIE imaging	Sensitive to TME	High; Theranostic SDT	[87], [93]

## 3 Tumor hypoxia alleviation

Tumor hypoxia limits availability of O_2_ and reduces ROS production, significantly compromising the SDT efficacy. To address this challenge, various strategies, categorized by O_2_-delivery and in-situ O_2_ generation, have been developed to enhance O_2_-supply and overcome hypoxia in the TME. These include the use of O_2_-carriers, such as PFC-based nanoparticles, hemoglobin (Hb) and O_2_-filled microbubbles (MBs), which can deliver O_2_ directly to tumor sites. The other approach is the use of H_2_O/H_2_O_2_ splitting systems to generate O_2_ in-situ within the tumor, thereby replenishing O_2_-levels. In the following, we will discuss the advantages of each strategy as well as the potential clinical translation.

### 3.1 O_2_-delivery for tumor reoxygenation

#### 3.1.1 O_2_-MBs

**Fig. 6:**
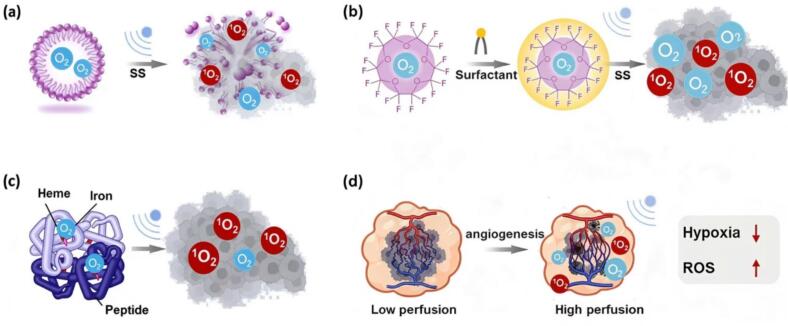
Schematic overview of O_2_-delivery strategies for reoxygenation enhanced SDT. (**a**) O_2_-MBs release O₂ upon US activation to boost ROS levels in TME; (**b**) PFC-based nanoparticles act as high-capacity O₂-reservoirs, enabling US-triggered release in TME; (**c**) Hb delivering O₂ to TME with enhanced ROS generation; (**d**) Normalization abnormal tumor vasculature restores perfusion, providing improved O_2_-delivery and SDT efficacy. SS is the abbreviation of sonosensitizer.

O_2_-encapsulated MBs provide a promising solution to overcome tumor hypoxia[103]. Biocompatible O_2_-MBs can be administered orally, via injection, or through intraperitoneal perfusion, making them a promising adjuvant for SDT. The O_2_-MBs can penetrate tumor tissues and release O_2_ under US activation. Locally increased O_2_-concentration would increase ^1^O_2_ production to achieve enhanced SDT efficacy (Fig. 6a) [104], [105], [106]. Various O_2_-carriers, including lipid-based, polymeric, protein-based, silica-based, or surfactant-based materials, can be used to formulate O_2_-MBs, which provide advantages for controlled release, stability, and biocompatibility in SDT[105], [107]. In a typical example, J Owen et al.[104] formulated O_2_-MBs by glycyrrhizic acid, lecithin, citric acid, and glycerol-based membrane with O_2_-encapsulation reaching a concentration of 3.2 mg/L. In a pancreatic cancer model, Rose Bengal based SDT demonstrated that the tumor growth rate in mice receiving O_2_-MBs was significantly lower compared to those without O_2_-delivery (Table 2). In addition, necroptosis caused by US-targeted nanobubble destruction has been proven to boost SDT immunotherapy, thus the synergistic effect further enhances therapeutic outcomes in breast cancer[108].

**Table 2:** O_2_-delivery strategies for reoxygenation enhanced SDT.

**Strategies**	**Carrier**	**Sonosensitizers**	**Animal model** **(tumor cell)**	**US** **(Freq./Power)**	**Dosage.** **(Concn.)**	**O_2_ Concn.**	**Cell mortality**	**Tumor inhibition**	**Ref.**
**O_2_-MBs oxygenation**	Liposome	RB	C.B-17-SCID mice (BxPc-3)	1.0 MHz, 3.5 W/cm^2^, 30 % duty, 3.5 mins	4 × 100 uL, 15d (1 mM)	3.2 mg/L	−	∼75 %	[104]
	Liposome	Ce6	BALB/c mice (4 T1)	1.0 MHz, 1.5 W/cm^2^	5 × 200 uL, 8 d (20 μg/100 μL)	6.48 mg/L in PBS	∼46 %	∼79 %	[108]
**PFC-based oxygenation**	PFH + Lipid+ DSPE-PEG	IR-780	BALB/c mice (4 T1)	1.0 MHz, 1.6 W/cm^2^, 30 % duty, 8 mins	3 × 200 ul, 6 d (2.5 mg/kg)	16.4 ± 2.1 mg/L	～40 %	～82 %	[117]
	PFH + Liposome	HMME	BALB/c mice (4 T1)	1.0 MHz, 1.6 W/cm^2^, 50 % duty, 5 mins	2 × 200 ul, 3 d (2 mg/kg)	−	～60 %	∼85 %	[137]
	PFOB+ DSPE-PEG+ DSPE-PEGNH_2_	Pyropheophorbide	NCI-N87 nude mice (NCI-N87)	1.0 MHz, 2 W/cm^2^, 50 % duty, 5 mins	5 × 200 ul, 12 d （8 mg/kg）	12.72 mg/L	∼80 %	∼80 %	[138]
	PFTBA+ PLGA	IR780	BALB/c mice (4 T1)	1.0 MHz, 2.0 W/cm^2^ 5 mins	7 × 75ul, 14 d (25 mg/kg)	18.0 mg/mL	78.2 %	∼78 %	[116]
	PEG-PFC block copolymer	TPP	BALB/c mice (Renca)	1.0 MHz, 2.5 W/cm^2^, 50 % duty, 5 mins	3 × 100 ul, 4 d (120 mg/kg)	4.3 mg/L	∼70 %	∼60 %	[118]
	PFSEA + PEG + PFCE COPs	THPP	Balb/c mice (CT26)	40 kHz, 2 W, 30 mins	1 × 5 mg/kg	6 mg/L	～70 %	～65 %	[121]
	PFC-organosilica	IR780	Nude mice (PANC-1)	1.0 MHz, 1.0 W/cm^2^, 100 % duty, 3 mins	3 × 33 mg/kg 13 d	∼14 mg/L	∼65 %	∼90 %	[120]
	PAsp(DET)+ PEG + PFH	PpIX	BALB/c mice (4 T1)	1.0 MHz, 2.6 W/cm^2^, 25 % duty, 5 mins	6 × 100 ul, 24d (6.25 mg/kg)	∼12 mg/L	∼65 %	∼100 %	[119]
**Protein-O_2_ delivery**	Hb	ZIF-8	BALB/c mice (4 T1)	1.0 MHz, 1.5 W/cm^2^, 50 % duty, 2 mins	7 × 100 ul, 14 d (10 mg/kg)	∼16 mg/mL	∼70 %	∼88 %	[127]
	HSA-Hb	MnPcS	BALB/c mice (4 T1)	1.0 MHz, 1 W/cm^2^, 50 % duty, 3 h	1 × 200 ul (600 μg/mL)	∼6 mg/L	∼70 %	∼80 %	[125]
**Vascular normalization**	ZA + M2pep+ liposome	HMME	BALB/c mice (4 T1)	2.0 W/cm^2^, 30 s	4.20 mg/kg, 16 d	−	∼ 90 %	90 %	[136]
	NO+ Lipid	Zinc phthalocyanine	BALB/c mice (Panc02)	1.0 MHz, 0.8 W/cm^2^, 50 % duty 1.0 MHz, 1.0 W/cm^2^ 100 % duty, 5 mins	2 × 200 μL, 3 d (ZnPc 0.09 mM)	−	91 %	∼100 %	[131]

Tumor-targeting O_2_-MBs realized by ligands functionalization could further enhance the reoxygenation. R Song et al.[105] designed O_2_-MBs coated with acetylated dextran (AC-DEX), a water-soluble polysaccharide dextran modified by its hydroxyls with acetal moieties to increase its hydrophobicity. Under neutral conditions, the intact polymer shell effectively prevented the early release of O_2_ during blood circulation. However, the acetyl group in AC-DEX undergoes hydrolysis in acidic TME to transform the shell into water-soluble glucan. This pH-responsive design enabled a spontaneous burst of O_2_-MBs only in TME, thus increasing its tumor-targeting ability. In vivo results showed that after injection into the CNE2 tumor-bearing mice, the O_2_ concentration in the tumor area gradually increased ∼ 6 times and reached a peak 2 h post- injection (Table 2). O_2_-MBs enhanced SDT cases summarized in Table 2 indicate that tailored O_2_-MBs is a feasible tool to alleviate tumor hypoxia and enhance therapeutic outcomes of SDT.

#### 3.1.2 PFC O_2_-carrier

PFC are synthetic compounds with unique physicochemical properties, characterized by high O_2_-solubility, biocompatibility, low toxicity and high biosafety[109]. Particularly, PFC demonstrates O_2_-solubility proportional to O_2_ partial pressure, independent of chemical bonding. At 25 °C and 1 atm, the O_2_-solubility in perfluorodecalin can reach approximately 40–50 mL O₂/100 mL, significantly higher than the 2.1 mL O₂/100 mL of water[110]. PFC are widely studied for alleviating tumor hypoxia, enhancing the efficacy of SDT in the form of nanoemulsions or MBs by using surfactants or polymeric coatings (Fig. 6b)[111]. Moreover, PFC-based emulsions such as Oxygent® is used as blood substitutes and have undergone clinical trials[112].

***PFC encapsulation***. So far, various liquid PFC derivatives have been utilized to formulate O_2_-carrying nanoparticles for tumor reoxygenation, including perfluorohexane (PFH), perfluoropentane (PFP), perfluorooctyl bromide (PFOB), perfluorotributylamine (PFTBA), and perfluoropolyether (PFPE), etc. (Table 2). Biocompatible polymers such as Pluronic[113] and lipids[114] are suitable candidates for the PFC encapsulation. By co-loading of sonosensitizers like Ce6 or HMME, the nanodroplets could effectively overcome tumor hypoxia and significantly boost ROS generation to enhance SDT efficacy. PFC-based reoxygenation bubbles can serve not only as O₂-carriers but also as vehicles for drugs or immune stimulators, thereby integrating SDT with chemotherapy or immunotherapy to synergistically enhance tumor-targeting, regression, and immune activation. Loading drugs like erlotinib and doxorubicin into PFC nanodroplets enabled a synergistic interaction between O₂-enhanced SDT and chemotherapy, thereby significantly improving the overall therapeutic efficacy[115], [116]. Besides, O_2_-filled PFH nanodroplets loaded with IR-780 and STING pathway agonist DMXAA could effectively synergize SDT and immune checkpoint blockade, thus enhancing tumor cell killing[117].

***PFC functional polymer***. Synthesis of PFC-containing polymers is another feasible strategy for constructing reoxygenation SDT nanocarriers (Table 2). Our group reported porphyrin-loaded PFC core nanoparticles self-assembled from PFC-PEG block copolymers prepared via RAFT polymerization[118]. The PFC nanoparticles showed enhanced O_2_-loading (4.3 mg/L, 20 % higher than control) and elevated intracellular ROS generation, which induced extensive necrosis and tumor suppress in Renca model SDT. Similarly, Zeng et al.[119] developed PFC-functional polymer nanovesicles via ring opening polymerization with protoporphyrin IX modification, enabling increased O_2_-loading in PFH matrices, thereby alleviating hypoxia and enhancing SDT efficacy. In a complementary top-down approach, Chen et al.[120] grafted fluorocarbon chains onto hollow mesoporous organosilica, producing O_2_-reservoir nanoplatforms. After IR780 loading, it directly delivered O_2_ to hypoxic pancreatic tumor and boosted ROS production under US irradiation. Furthermore, PFC functionalization and encapsulation have been shown to act synergistically. Yang et al.[121] constructed fluorinated covalent polymer frameworks (THPPpf-COPs), which further encapsulated PFCE to provided dual O_2_-reservoir. Upon US activation, the nanosystems significantly enhanced ROS production and induced immunogenic cell death, highlighting their potential in finely engineered SDT nanomedicine.

#### 3.1.3 Protein O_2_-carrier

Hemoglobin (Hb) is widely used in O_2_-carrying nanoparticles due to its natural O_2_-binding and release properties, as well as its good biocompatibility[122]. Natural biocompatibility and low immunogenicity of Hb allow it to circulate in the body for a long period with minimal adverse reactions. Hb has a tetrameric structure, with each subunit containing a heme group. The Fe^2^⁺ at the center of the heme group can reversibly bind to O_2_, allowing each Hb molecule to carry up to four O_2_ molecules (Fig. 6c). Additionally, Hb exhibits a cooperative effect, when one subunit binds to O_2_, the affinity of the other subunits for O_2_ increases, thereby enhancing the O_2_-loading efficiency[123]. Importantly, the binding of Hb to O_2_ is reversible, allowing it to bind O_2_ in high-O_2_ environments and release O_2_ in low-O_2_ or acidic environments, making it particularly suitable for alleviating hypoxic TME[124].

In a typical example, Mn-phthalocyanine (MnPcS) was encapsulated into a human serum albumin-Hb hybrid protein nanosystem (MnPcS@HPO) at a loading efficiency of ∼ 84.7 %, providing spherical nanoparticles of ∼ 75 nm[125]. After O_2_ saturation and administration, MnPcS@HPO released O₂ selectively in hypoxic TME and generated significantly higher ^1^O_2_ compared to controls. The reoxygenation strategy leads to a cell death rate (∼75 %) higher than that without Hb support (∼55 %), and markedly inhibited 4 T1 tumor growth in vivo under US. Similarly, HMME was loaded into a ZIF-8 framework and modified with Pluronic F127 and Hb to construct HFH@ZIF-8, with an encapsulation efficiency of ∼ 69 % and particle size of ∼ 91 nm[126]. The Hb shell maintained O_2_-carrying function, as confirmed by oxy-/deoxyhemoglobin spectral shifts. After O_2_– binding, HFH@ZIF-8 + O₂ induced strong ROS production and almost complete eradication of MRSA colonies in vitro, while in vivo it significantly reduced myositis abscess area and bacterial load. More strikingly, Hb itself was validated as a natural metalloporphyrin sonosensitizer[127]. Without extra sonosensitizers, O₂@Hb@ZIF-8 (OHZ) nanoparticles achieved pH-responsive Hb/O₂ release (1.6-fold higher O₂ release at pH 5.5 than O₂@ZIF-8), and robust ^1^O_2_ generation at a high rate. In 4 T1 tumor models, OHZ nanoparticles triggered severe mitochondrial dysfunction and apoptosis, leading to strong tumor suppression with minimal systemic toxicity. Collectively, these Hb-based nanosystems (Table 2) demonstrated the dual role of Hb as both an O_2_-carrier and sonosensitizer, while integration with protein or MOF carriers provides stability, tumor targeting, and controlled O_2_-release. By effectively alleviating hypoxia and amplifying ROS production, such platforms have achieved striking therapeutic efficacy in both cancer and infection models, underscoring the translational promise of Hb-based reoxygenation SDT.

#### 3.1.4 Tumor vascular normalization

Tumor vasculature is often characterized by irregular endothelial cell arrangement, increased vessel permeability, and poorly organized blood vessel networks[128]. These vascular abnormalities lead to poor perfusion and insufficient O_2_ diffusion of the tumor solid and aggregate hypoxia[129], [130], which impairs the efficacy of O_2_-dependent SDT. Thus, tumor vascular normalization is a feasible strategy to increase O_2_-supply in tumor solid and enhance the ROS generation as well as SDT efficacy (Fig. 6d).

Nitric oxide (NO) has been explored for tumor vessel normalization. Fang et al. developed a US-responsive lipid nanosonosensitizer (IR&ZnPc@LNP-NO), co-loaded with the sonosensitizer zinc phthalocyanine, IR, and an NO donor in lipid nanoparticles[131]. Release of NO remodeled the TME by normalizing vasculature and degrading dense extracellular matrix. The cascade strategy-TME remodeling, ROS enhanced SDT and multimodal therapy effectively overcomes stromal and immunosuppressive barriers, offering a robust platform for treating deep-seated pancreatic cancer. Tumor-associated macrophages (TAMs) also play a key role in angiogenesis, with M2-type TAMs driving VEGF secretion and abnormal vessel formation[132], [133], [134], [135].To address this, Wang et al.[136] designed M−H@lip-ZA, a nanoliposome encapsulating zoledronic acid (ZA) in the core, hematoporphyrin monomethyl ether (HMME) in the lipid bilayer, and M2-targeting peptide (M2pep) on the surface. ZA-mediated M2 TAM depletion induced marked tumor remodeling: M2-like TAMs death rates exceeded 90 %, and significant vascular normalization. In the 4 T1 tumor-bearing mouse model, vasculature normalized group achieved a tumor suppression rate of 90 %, significantly outperforming controls (Table 2).

#### 3.1.5 Comparison of O_2_-delivery strategies

Comparing all the O_2_-delivery strategies, O_2_-MBs offer US-triggered, site-specific O_2_-release, enabling simultaneous O_2_-delivery for SDT enhancement with real-time US imaging and high biosafety. However, their short circulation time (<24  h) and potential O_2_-leakage necessitate repeated administration. PFC-based carriers possess highest O_2_-loading capacity (Table 2) and considerable tumor inhibition rates between 60–95 %; yet their slow clearance and synthetic complexity raise concerns about chronic toxicity and manufacturing cost. Hb mimic systems are clinically translatable due to excellent biocompatibility and modifiability with targeting ligands, but suffer from low O_2_ capacity (1/10 that of PFC), protease sensitivity, and cold-chain storage requirements. Vascular normalization strategies using agents like ZA offer long-lasting hypoxia relief and immunotherapy synergy and provide superior cell mortality and tumor clearance capability in SDT (Table 2), though they have delayed onset (∼7 days) and risk systemic toxicity.

### 3.2 In-situ O_2_-generation

**Fig. 7:**
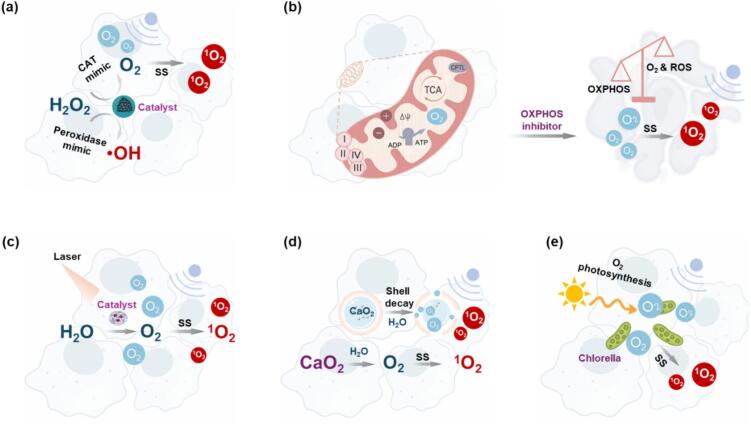
Schematic overview of of in-situ O_2_-generation for tumor reoxygenation enhanced SDT. (**a**) Endogenous H₂O₂ decomposition via catalase (CAT) and peroxidase mimics generates O_2_ and •OH, boosting both type I and type II ROS production; (**b**) Inhibition of mitochondrial oxidative phosphorylation (OXPHOS) reduces cellular O_2_-consumption for enhanced ROS generation; (**c**) Photocatalytic H_2_O_2_ splitting produces O₂ in-situ, improving O_2_-supply and ROS yield. (**d**) Decomposition of solid peroxides (e.g., CaO₂) releases O₂ under physiological conditions; (**e**) Photosynthetic microorganisms generate O₂ to alleviate tumor hypoxia and promote ROS generation.

External O_2_-delivery often fails to uniformly oxygenate the tumor, since hypoxia is heterogeneous across different regions. In-situ O_2_-generation, where O_2_ is produced within the TME, is gaining increasing attention as a promising alternative. Several strategies (Fig. 7) including catalytic endogenous H₂O₂ decomposition[139]; reduction of cellular O_2_-consumption[140]; O_2_-releasing peroxides[141] and photosynthetic O_2_-generation[142], have been developed for in-situ O_2_-generation.

#### 3.2.1 Endogenous H_2_O_2_ decomposition

Tumor cells exhibit elevated H₂O₂ levels due to abnormal metabolism and disrupted redox homeostasis, mainly arising from mitochondrial respiration, NADPH oxidase, and peroxidase activity[143]. While excessive H₂O₂ promotes tumor progression, it also provides a substrate for in-situ O₂ generation to relieve hypoxia and enhance SDT efficacy. CAT is an effective antioxidant enzyme that decomposes H₂O₂ into O₂, thereby increasing intratumoral oxygenation and amplifying ROS production during SDT (Fig. 7a). In a typical example, Liu et al.[144] designed a thermo-triggered chitosan/β-glycerol phosphate (CS/GP) hydrogel system with CAT and sonosensitizer TCPP conjugated. After intratumoral injection, the precursor solution underwent sol–gel transition at body temperature, enabling localized retention of TCPP-CAT. The CAT loaded hydrogel continuously decomposed H₂O₂ into O₂ to promote ^1^O₂ generation under US and sustained structural stability for repeated treatments. This “single-dose, multiple-treatment” platform significantly improved SDT efficacy (Table 3). However, CAT faces challenges of high cost, instability under physiological conditions, and sensitivity to proteases[8]. To address these limitations, nanomaterials with intrinsic catalase-like activity have emerged as robust alternatives. Compared with CAT, nanozymes offer superior stability, facile preparation, and tunable functionality. Materials such as MnO₂ and COF-based nanosystems have been engineered to efficiently decompose H₂O₂ to alleviate tumor hypoxia, thereby representing a promising strategy to strengthen reoxygenation enhanced SDT (Table 3).

**Table 3:** In-situ O2-generation for enhanced SDT.

**Approach**	**Catalyst** **/Inhibitor**	**Sonosensitizers**	**Animal model** **(tumor)**	**US** **(Freq./Power)**	**Dosage freq.** **/Concn.**	**Tumor oxygenation**	**Cell mortality**	**Tumor inhibition**	**Ref.**
**Endogenous H_2_O_2_ decomposition**	MnO_2_	Ce6	BALB/c mice (PC-3)	1 W/cm^2^, 10 mins	3 × 5 mg/kg	8 mg/L	76.4 %	∼75 %	[155]
	CAT	Ce6	BALB/c mice (4 T1)	1.0 MHz, 1.5 W/cm^2^, 50 % duty, 5 mins	3 × 5 mg/kg, 3 d	4 mg/L	∼80 %	∼86 %	[156]
	CAT	TCPP	BALB/c mice (4 T1)	1.0 MHz, 1.5 W/cm^2^ 3 mins	1 × 100 μL 1 d (2 mg/mL)	15.3 mg/L	61.2 %	∼78 %	[157]
	Mn^2+^	Mn-porphyrin	BALB/c mice (4 T1)	2 W/cm^2^, 50 % duty 5 mins	3× /μL 5 d	16 mg/L	66.25 %	∼100 %	[158]
	MOF	IR780 CD	BALB/c mice (4 T1)	30 kHz, 2.0 W/cm^2^, 5 mins	3 × 10 mg/kg, 5 d	−	93.10 %	68.10 %	[159]
	MnO_2_	AIPH	Balb/c mice (SKOV-3)	2.0 W/cm^2^ 5 mins	3 × 100 μL 5 d (2.1 mg/mL)	12 mg/L	70.20 %	97 %	[160]
	CAT	TCPP	BALB/c mice (CT26)	40 kHz, 3 W/cm^2^	1 × 25 μL 1 d (5 mg/kg)	11 mg/L	50 %	∼100 %	[144]
	Fe(II)/Fe(III)	PpIX	BALB/c mice (Saos-2)	1.0 MHz, 1.4 W/cm^2^	2.0 mg/kg	9 mg/L	60 %	94.9 %	[145]
	Mn^2+^	Ce6	Athymic nude mouse (HepG2)	1.0 MHz,1.5 W/cm^2^ 50 % duty, 3 mins	1 × 100 μL 1 d (5 mg/kg)	6 mg/L	∼70 %	91 %	[161]
	MnO_2_	Cur	/ (HepG2)	1 W/cm^2^, 1 min	6 × 5 mg/kg 2 d	18 mg/L	∼85 %	99 %	[162]
	MnO_x_	PpIX	BALB/c mice (U87)	1 MHz, 2.5 W/cm^2^, 3 mins	1.0 mg/kg	7 mg/L	∼85 %	96 %	[163]
	MnO_2_	HMME	BALB/c mice (MCF-7)	1.0 MHz, 1.5 W/cm^2^, 50 % duty, 1 min	1 × 100 μL 1 d (5 mg·kg–1)	28 mg/L	77.20 %	96 %	[164]
	MnO_2_	Ce6	BALB/c mice (4 T1)	10 W/cm^2^, 5 mins	1 × 200 μL 1 d (200 µM)	19.2 mg/mL	78 %	∼100 %	[165]
**Mitochondria dysfunction drugs**	ATO	HB	Balb/c mice (HeLa)	1.0 MHz, 1.5 W/cm^2^, 6 mins	1 x 100 µL 1 d (20 mg/kg)	−	41.7 %, 9.7 % (with PDT)	∼95 %	[148]
	Metformin	IR780	Balb/cmice (MDA-MB-231)	1.0 MHz, 2.5 W/cm^2^, 5 mins	1 × 200 μL 1 d (2 mg/kg)	−	30 %	∼ 85 %	[139]
	Vanadium-based nanozyme (VOx)	ICG	Balb/c mice (4 T1)	1.0 W/cm^2^, 5 mins	1 d (20 mg/kg)	−	33.43 %	∼65 %	[166]
	Metformin	ICG	Balb/c mice (4 T1)	1.0 MHz, 2.0 W/cm^2^, 5 mins	3 × 100 μL 5 d (2.1 mg/mL)	−	30 %	∼70 %	[167]
	ATO	PFODBT	/(4 T1)	1.0 W/cm^2^, 10 mins	1 d (20 μg/mL)	−	57.9 ± 0.7 %	＞95 %	[168]
	CO	TPPS	Balb/c mice (4 T1)	1.0 MHz, 0.56 W/cm^2^, 50 % duty, 5mins	4 × 1 mg/kg 6 d	−	75.1 %	89.88 %	[169]
	3-Bromopyruvate (3BP)	IR780	Balb/c mice (Panc02)	1.0 MHz, 0.5 W/cm^2^, 50 % duty, 2.5mins	5 × 1 mg/mL 8 d	OCR decreased ∼ 74 %	∼ 50 %	∼ 50 %	[170]
**H_2_O_2_ splitting**	C3N4	Ce6	C57BL mice (B16-F0)	1 kHz, 50 % duty	21d (5 mg/kg)	DO increased to 32 %, up from 26 % in the control group	∼76 %	∼75 % .	[150]
	Mesocrystalline Zinc Sulfide	mZnS	/(4 T1)	1 MHz, 3 W/cm^2^, 10 % duty,10 mins	30d (30 mg/kg)	7.9 μmol/min/mg	∼76 %	∼76 %	[171]
**Peroxides decomposition**	CaO₂	RB	/(BxPC-3)	1 MHz, 3 W/cm^2^, 30 % duty, 3.5 mins	14d (1.0 μg/mL）	5 mg/L	∼75 %	∼70 %	[141]
**Photosynthesis**	PCC 7942	Ce6	/(4 T1)	1 MHz, 1.5 W/cm^2^, 50 % duty, 5 mins	14d (500 µg/mL)	40 μmol/L	85 %	92.2 %	[152]
	MChl	HP	/(B16)	650 kHz, 0.5 W/cm^2^, 1 min	2 × 2 mg/kg CQ + 0.95 mg/kg HP, 6 d	2.62 mg/L	88 %	87 %	[154]

Beyond type II ROS enhancement, H₂O₂ also serves as a precursor for type I ROS. Under metal catalysis and US activation, H₂O₂ yields HOO• and HO• radicals to induce DNA damage, lipid peroxidation, and membrane disruption, thereby intensifying tumor cell death. Leveraging this, Hao’s group developed mesoporous organosilica with ferrate (IV) and protoporphyrin IX embedded, integrating catalase-,and peroxidase-like activities[145]. In this system, ferrate (IV) decomposed H₂O₂ into O₂ and HOO•/HO• radicals to alleviate hypoxia and amplified type I and II ROS generation simultaneously. This catalytic cascade resulted in superior in vivo SDT efficacy with a tumor suppression efficiency of 94.9 %. It demonstrated the potential of integrated catalytic nanoplatforms to synergistically regulate hypoxia and boost both type I and II ROS for robust tumor eradication.

#### 3.2.2 Mitochondria dysfunction reduce O_2_-consumption

Reducing tumor O_2_-consumption is an effective strategy to increase ROS production for SDT efficacy enhancement[146]. Since OXPHOS consumes O_2_ as the terminal electron acceptor in the electron transport chain (ETC), inhibiting OXPHOS spares intratumoral O_2_ for ROS generation (Fig. 7b). Agents such as metformin, atovaquone (ATO), and oligomycin can block ETC complexes or ATP synthesis, thereby reducing O₂ demand and amplifying SDT outcomes[147].

Building on this concept, Liu’s group designed a multifunctional nanoplatform (PHAR) composed of polydopamine core-mesoporous and silica shell nanoparticles functionalized with RGD peptide for tumor targeting, co-loaded with ATO and the sonosensitizer chlorin e6-C-15-ethyl ester (HB)[148]. By releasing ATO, PHAR attenuated mitochondrial OXPHOS that verified by JC-1 staining of disrupted membrane potential and increased ROS generation under US. In vitro, PHAR + US reduced cell viability to 41.7 %, which further dropped to 9.7 % when combined with PDT. In vivo, PHAR achieved complete tumor suppression without recurrence throughout the treatment period, demonstrating the power of OXPHOS inhibition in relieving hypoxia and amplifying ROS-driven SDT (Table 3). Similarly, Zhang’s group formulated pH-responsive liposomes encapsulating IR780 and metformin. While IR780 provided sonosensitization, metformin inhibited complex I of the ETC, reducing ATP production and O_2_-consumption[139]. Co-loading of metformin significantly increased the ROS level and delayed tumor growth after US irradiation (Table 3). These results confirm that mitochondrial respiration inhibition can effectively reoxygenate tumors, strengthen ROS production, and enhance SDT efficacy.

#### 3.2.3 Catalytic H_2_O splitting

H_2_O splitting into O₂ within the TME has recently emerged as another promising strategy to relieve tumor hypoxia for enhanced SDT (Fig. 7c)[149]. Abundant H_2_O in tumor tissue offers a continuous and renewable O_2_-source. Zhang et al. designed UCNPs-C3N4-Ce6 nanoplatforms, where C3N4 photocatalysts were assembled on upconversion nanoparticles to suppress electron-hole recombination, thereby improving H_2_O-splitting efficiency[150]. Under light irradiation, this system dissolved O_2_ to 32 % from 26 % of controls to alleviate tumor hypoxia. When combined with Ce6 under US, ^1^O₂ generation was significantly boosted with obvious B16-F0 tumor growth inhibition (Table 3). While photocatalytic H_2_O-splitting is suitable for superficial tumors accessible to light, its penetration depth is limited. Whereas sonocatalytic H_2_O-splitting uses US-induced cavitation to generate •OH and O₂ without light, offering superior performance in deep tumors. Importantly, integrating both modalities in photo-sonocatalytic systems could couple the high efficiency of light-driven catalysis with the deep penetration of US, providing a synergistic O_2_-supply to overcome hypoxia and maximize SDT efficacy.

#### 3.2.4 Exogenous peroxides decomposition

Exogenous peroxides such as CaO₂, Na₂O₂, and MgO₂ offer high O_2_ yields by decomposing in H_2_O, thereby amplifying ROS generation to enhance SDT. Nicholas et al. designed CaO₂ nanoparticles that hydrolyze inside tumor tissue to simultaneously produce H₂O₂ and O₂[141]. To prevent uncontrolled O₂ bursts, pH-sensitive methacrylate polymers were used to coat CaO₂ nanoparticles, ensuring stability at neutral pH and controlled degradation in acidic TME conditions (Fig. 7d)[151]. By loading with the sonosensitizer Rose Bengal, nanoparticles raised intratumoral O₂ partial pressure within 3 mins and significantly reduced BxPC3 pancreatic cancer cell viability under US activation in vitro (Table 3)[141]. In tumor-bearing mice, CaO_2_ loaded nanoparticles significantly inhibited tumor growth around 70 %, whereas controls and RBcNPs alone showed negligible effects. This case highlighted the effectiveness of the exogenous peroxides-based tumor reoxygenation strategy for SDT efficacy enhancement.

#### 3.2.5 Photosynthesis

Photosynthesis offers an innovative and self-sustained strategy for tumor reoxygenation by generating O₂ within TME using light-driven microorganism synthesis. Thus, synergizing photosynthetic oxygenation with SDT can alleviate hypoxia and amplify ROS production (Fig. 7e)[142]. Cyanobacteria (Cyan) have been widely explored for reoxygenation enhanced SDT. Yang et al. developed a hybrid platform integrating Ce6 sonosensitizers with Cyan[152]. Ce6 not only acted as a sonosensitizer but also a light source, that generates sonoafterglow luminescence under US, to stimulate Cyan photosynthesis-based O₂-production. Through high O₂-output and elevated ^1^O₂ generation, this system alleviated hypoxia in 4 T1 cells as proven by reduced HIF-1α expression and [Ru(dpp)₃] Cl₂ staining. In vivo tumor suppression rates reached 92.2 % without shielding and 86.0 % with a 3-mm tissue barrier, highlighting robust sonophotosynthetic oxygenation effect in SDT enhancement (Table 3).

As a safer alternative, Chlorella-based systems harness its chlorophyll-rich photosynthetic machinery to produce O_2_ in-situ, supporting tumor reoxygenation under light stimulation[153]. Gao et al. engineered macrophage-mimetic microalgae conjugated with sonosensitizer- and drug-loaded liposomes (MChl-CQ-HP-NP)[154]. Under light irradiation, Chlorella-mediated O_2_-production alleviated hypoxia in B16 melanoma cells, as demonstrated by reduced HIF-1α fluorescence. Combination therapy integrating light-driven oxygenation, SDT, and autophagy inhibition achieved potent cytotoxicity in vitro and significant tumor regression in vivo (Table 3). Together, cyanobacteria- and algae-based photosynthetic systems exemplify a powerful class of reoxygenation strategies that, when coupled with SDT, achieve efficient hypoxia relief and markedly improved antitumor efficacy.

#### 3.2.6 Comparison of in-situ O_2_-generation strategies

Endogenous H₂O₂ decomposition, catalyzed by CAT or nanozymes enables targeted intratumoral O₂-generation in TME, offering minimal side effects and synergistic benefits for SDT and immune modulation. However, its efficacy depends on sufficient intratumoral H₂O₂ concentrations. Mitochondrial respiration inhibitors enhance ROS generation by both reducing O₂-consumption and catalyzing H₂O₂ generation but may chemotherapy drugs pose off-target toxicity to normal cells. Photocatalytic H_2_O-splitting provides continuous O_2_-supply independent of the TME but is limited by external light requirements and shallow tissue penetration. Solid peroxides like CaO₂ deliver high O₂ output but lack targeting, risk oxidative stress in normal tissues, and offer poor control. Photosynthesis strategies provide sustained O₂-supply but face challenges in in vivo implementation due to light and substrate limitations. Among these, endogenous H₂O₂ decomposition stands out for its clinical potential with tumor eradication rates higher than the other strategies (Table 3). Besides, with mature platforms such as injectable hydrogels, long-term effectivity, balanced safety, enhanced therapeutic efficacy could be achieved from a single dose.

## 4 Challenges and future perspectives

Despite the promising advances in organic sonosensitizers and O_2_-modulation strategies for SDT, several critical barriers must be overcome to develope advanced SDT and enable the clinical translation. These challenges are rooted in the complexity of acoustic-chemical interactions, limited biological mechanistic clarity, and the preclinical nature of most studies. Currently, the vast majority of investigations remain at the in vitro or small animal level, often lacking robust pharmacokinetic, biodistribution, and toxicity data necessary for human application.To bridge these gaps, future research should prioritize:

### 4.1 Mechanistic elucidation of sonoluminescence and its interaction with organic sonosensitizers

Understanding the molecular mechanism of ROS generation in SDT is essential for rational sonosensitizer design. Future efforts should focus on direct detection of sonoluminescence-triggered excited-state transitions and electron transfer pathways using spectroscopic and computational tools. Clarifying how US parameters interact with sensitizer structure will enable the engineering of more efficient and selective ROS generators.

### 4.2 Development of ROS-selective probes and cell death mapping

Accurately characterizing and quantifying ROS species and their downstream biological effects remains a challenge. Designing selective probes for ^1^O_2_, •OH, and •O_2_^–^, alongside high-resolution tracking of cell death pathways (e.g., apoptosis, ferroptosis), will help correlate sonosensitizer performance with therapeutic outcomes and support optimization for immunogenic effects.

### 4.3 Smart nanoplatforms for O_2_-responsive SDT

Multifunctional nanoplatforms that integrate O_2_-generation (e.g., nanozymes, peroxides, photocatalysts), controlled drug release, and real-time imaging offer a path toward precision SDT. Emphasis should be placed on US-responsive systems that couple on-demand O_2_ with ROS burst, ideally using biodegradable or clinically approved carriers for improved safety and scalability.

### 4.4 Clinical translation and regulatory readiness

To accelerate clinical adoption, future research should prioritize components with established safety (e.g., porphyrin derivatives, PFC carriers), validate efficacy in immunocompetent models, and define pharmacokinetics and biodistribution under therapeutic US. Collaboration with clinicians to establish regulatory standards and SDT-specific endpoints will be essential for translational success.

## 5 Conclusion

Organic sonosensitizers hold significant promise for non-invasive and precise cancer therapy via SDT. This review systematically outlines two primary axes of advancement: (1) the molecular engineering of organic sonosensitizers to enhance ROS generation via strategies such as heavy atom substitution, D-A modulation, and solubility enhancement, etc.; and (2) hypoxia alleviation through O_2_-delivery and in-situ O_2_-generation approaches, including O_2_-MBs, PFC nanodroplets, vascular normalization, OXPHOS inhibition, and endogenous H₂O₂ catalysis. Across these domains, we critically compared strategies in terms of ROS enhancement, safety, scalability, and clinical potential. Notably, porphyrin-based sensitizers and PFC carriers emerged as promising clinically translatable candidates. Moreover, integrating O_2_-modulation with smart drug delivery and immune activation offers a unified path forward for next-generation SDT. However, the field must now pivot toward addressing acoustic-chemical mechanistic, clarifiying ROS-based cell death mapping, developing biocompatible multifunctional platforms, and navigating regulatory challenges. By aligning basic science discoveries with clinical development priorities, future SDT systems can achieve precise, safe, and effective oncologic outcomes—moving closer to real-world therapeutic application.

## CRediT authorship contribution statement

**Qianyun Shan:** Writing – review & editing, Writing – original draft. **Rumei Li:** Writing – original draft. **Bin Ying:** Writing – original draft. **Wei Zhu:** Writing – original draft. **Xiaojin Wu:** Resources. **Shouxing Xu:** Resources. **Xuanxuan Zhang:** Software. **Zhikang Xu:** Software. **Xinyue Zhu:** Resources. **Weiyu Chen:** Writing – review & editing. **Kai Zhang:** Writing – review & editing, Conceptualization. **Jian Chen:** Writing – review & editing, Supervision, Resources.

## Declaration of competing interest

The authors declare that they have no known competing financial interests or personal relationships that could have appeared to influence the work reported in this paper.
